# SIRT1 in B[a]P-induced lung tumorigenesis

**DOI:** 10.18632/oncotarget.4729

**Published:** 2015-08-04

**Authors:** Jianyi Lu, Min Zhang, Zhiyong Huang, Sufang Sun, Yongliang Zhang, Lei Zhang, Lirong Peng, Ailing Ma, Pan Ji, Jia Dai, Tong Cui, Heping Liu, Jimin Gao

**Affiliations:** ^1^ Zhejiang Provincial Key Laboratory for Technology & Application of Model Organisms, School of Life Sciences, Wenzhou Medical University, Wenzhou, Zhejiang, 325035, China; ^2^ Department of Cardiothoracic Surgery, Nanfang Hospital, Southern Medical University, Guangzhou, Guangdong, 510515, China

**Keywords:** B[a]P, SIRT1, TNF-α, β-catenin, lung cancer

## Abstract

Benzo[a]pyrene (B[a]P) is a carcinogen in cigarette smoke. We found that B[a]P induced SIRT1 in human bronchial epithelial BEAS-2B cell. SIRT1 was overexpressed in the lung of B[a]P-exposed mice and in human lung cancer biopsies. SIRT1 up-regulated TNF-α and β-catenin and down-regulated the membrane fraction of E-cadherin. In addition, SIRT1 promoted invasion, migration and tumorigenesis of BEAS-2B cells in nude mice upon B[a]P exposure. Thus, SIRT1 is involved in B[a]P-induced transformation associated with activation of the TNF-α/β-catenin axis and is as a potential therapeutic target for lung cancer.

## INTRODUCTION

Lung cancer is one of the most prevalent and lethal cancers all over the world. It accounts for 28% of all cancer related deaths and 14% of all new cancer cases annually [1]. Increasing amounts of epidemiologic data have indicated that cigarette smoking is the major cause of lung cancer [2–4]. Cigarette smoke is estimated to contain as many as 4000 chemicals [5, 6]. Of these chemicals, B[a]P, a prototypical polycyclic aromatic hydrocarbon (PAH) found at high concentrations in cigarette smoke, is the most important and strongest lung carcinogen [7–10]. Many studies demonstrate that B[a]P is responsible for both lung inflammation and lung cancer development [11, 12], but the underlying mechanisms have not been well elucidated.

SIRT1 also known as NAD-dependent deacetylase sirtuin-1, may function as an intracellular regulatory protein. Current researches focus on the biological functions of SIRT1 in obesity associated metabolic diseases, cancer, aging, cellular senescence, inflammatory signaling in response to environmental stress, development and placental cell survival [13–16]. SIRT1 also has an apparent (albeit context- and tissue type-dependent) role in tumorigenesis, mainly acting through its deacetylation of the tumor suppressor gene products such as p53 and Rb [17, 18]. SIRT1 showed increased expression in some types of cancers [19–21]. In some inflammation studies, researchers demonstrated that SIRT1 protected cells against chronic inflammation by controlling the acetylation of nuclear factor kappa B (NF-кB), a transcription factor involved in the innate immune response [22, 23]. In addition, SIRT1 protected dramatically from liver carcinogenesis not only by diminishing the inflammatory response, but also by protecting from the initial acute DNA damage triggered by ditehylnitrosamine [24]. Resveratrol, an activator of SIRT1 suppressed colitis-induced inflammatory makers(iNOS, COX-2, TNF-α) [25].

TNF-α is a significant cytokine involved in inflammation, immunity, cell migration/invasion and tumor progression. Our previous studies demonstrate that TNF-α is an essential inflammatory mediator for cell neoplastic transformation upon B[a]P/B[a]PDE exposure [26]. TNF-α might affect all tumorigenic steps by regulating inflammatory response, including proliferation, angiogenesis, invasion and metastasis, but the detailed mechanisms remain elusive [27–30].

β-catenin, the key factor of Wnt signaling pathway, is involved in tumorigenesis, such as lung cancer, ovarian and colon cancer. Clevers HC, *et al*. identified three putative β-catenin homologues in C. elegans and found that the functions of β-catenin in adhesion and in signaling were carried out by separate proteins [31]. During carcinogenesis, increased transcriptional activity of β-catenin correlated with the loss of E-cadherin-mediated cell adhesion and the increase of cell migration [32, 33]. Yoon Y *et al*. found that β-catenin positively regulated NF-кB activity, as well as the expression of inflammatory cytokines, including TNF-α, IL-6, IL-8 in lipopolysaccharide (LPS)-treated bronchial epithelial cells [34]. In prostate cells, acute TNF-α exposure disrupted β-catenin-E-cadherin interaction as well as cell migration [35].

To elucidate the role of SIRT1 in B[a]P-induced lung cancer, we studied the SIRT1 expression and functions in B[a]P-induced BEAS-2B cells, mice lung and patients’ lung biopsies. Our results indicated that SIRT1 was involved in the crosstalk between TNF-α and β-catenin and acted as a key regulator in cancer invasion and migration. Thus SIRT1 was responsible for B[a]P-induced sustained inflammation and lung cancer development.

## RESULTS

### SIRT1 expression was up-regulated in human lung cancer biopsies

**Figure 1:**
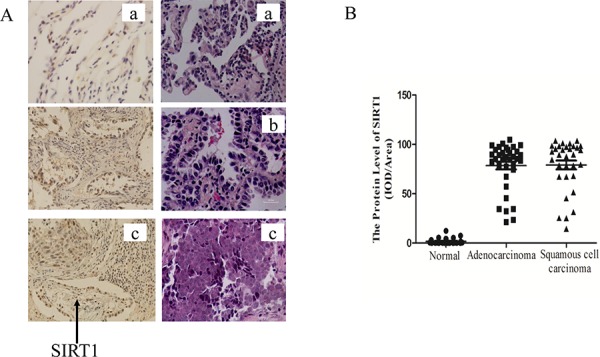
SIRT1 was up-regulated in human lung cancer biopsies **A.** Representative immunostaining of SIRT1 and HE staining in human lung biopsies. (a) Normal lung tissues, (b) Lung adenocarcinoma biopsies and (c) Lung squamous cell carcinoma biopsies. Magnification times:400×. **B.** SIRT1 staining integrated optical density (IOD) of human normal lung biopsies against cancer biopsies.

Several studies found that SIRT1 expression was up-regulated in various cancers such as leukemia, prostate cancer, skin cancer and colon cancer [19, 20, 36–38]. To determine the SIRT1 protein levels in human lung cancer tissue, we acquired the lung tissue biopsies from the First Affiliated Hospital of Wenzhou Medical University, including 33 normal biopsies, 36 lung adenocarcinoma and 32 lung squamous cell carcinoma biopsies. The samples were subject to *he*matoxylin-eosin (HE) *staining* and the immunohistochemical analysis to determine the SIRT1 protein levels. Representative immunostaining results of SIRT1 expression in normal and malignant lung biopsies were shown in Figure 1A. The SIRT1 protein expression levels in lung cancer biopsies, both adenocarcinoma and squamous cell carcinoma biopsies, were significantly higher than those in normal ones (*p* < 0.001) (Table 1 and Figure 1B). Therefore, SIRT1 expression was significantly up-regulated in lung cancer.

**Table 1:** SIRT1 expression levels in human normal lung and cancer biospies

Group	Cases	SIRT1 staining IOD(×10^4^)^a^
**Normal**	33	1.02 ± 1.95
**Adenocarcinoma**	36	80.48 ± 23.63***^b^
**Squamous cell carcinoma**	32	78.86 ± 26.59***^b^

a The SIRT1 staining integrated optical density(IOD) was measure by the Image Pro Plus analysis software. Data were semi-quantitatively analyzed and expressed as the mean value ± S.D.

b ***p < 0.001 was considered statistically significant.

### B[a]P induced SIRT1 expression in BEAS-2B cells

**Figure 2:**
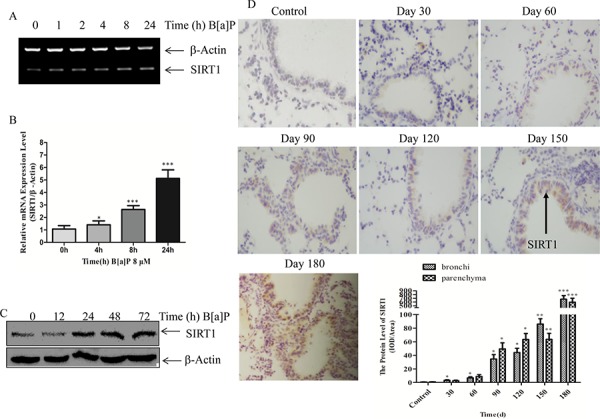

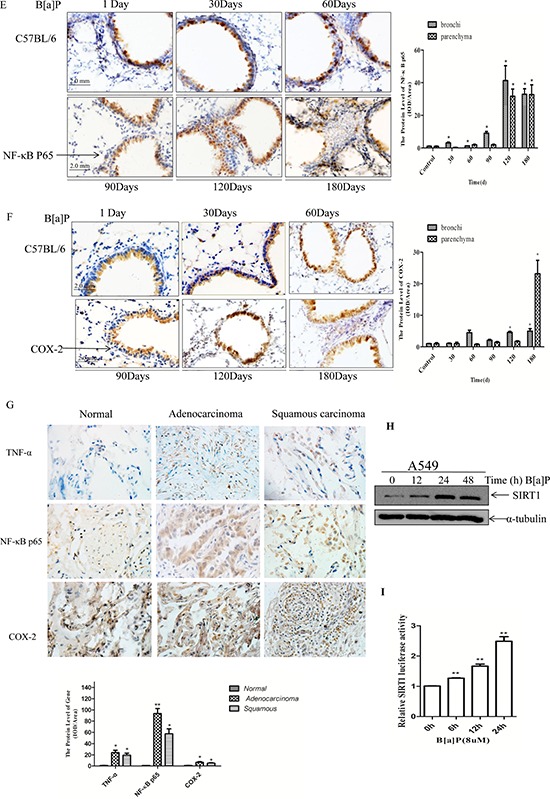
B[a]P induced SIRT1 expression in BEAS-2B cells and in mice lung biospies **A.** 2 × 10^5^ BEAS-2B cells in 6-well plates were treated with 8 μM B[a]P for various time as indicated. Total RNA were extracted with Trizol for semi-quantitative RT-PCR; and **B.** Real-time PCR assay. The results were expressed as the ratios of the SIRT1 induction relative to the medium control (relative SIRT1 induction). Each bar indicated the mean and standard deviation of the triplicates. **C.** BEAS-2B cells (2 × 10^5^ per well) in 6-well plates were cultured in 10% FBS DMEM at 37°C. When the cell density reached 60%∼70% confluence, the cells were exposed to 8 μM B[a]P from 0 to 72 h. Then cellular lysates were prepared for Western blotting using SIRT1 and β-Actin antibodies, respectively. **D–F.** Mice Lung sections were immunostained with the SIRT1/NF-кB P65/COX-2 antibodies and developed with DAB as described in the materials and methods. Magnification times:200×. IOD of parenchyma and bronchi were shown respectively. **G.** Representative immunostaining of NF-кB P65/TNF-α/COX-2 in normal human lung biopsies, lung adenocarcinoma biopsies and lung squamous cell carcinoma biopsies. Magnification times:200×. IOD of total lung tissue were calculated. **H.** Western blotting of SIRT1 in A549 cells at 0, 12, 24, 48 h under B[a]P treatment. **I.** The transcription activities of SIRT1 determined by dual luciferase report system. Results were expressed as mean ± S.D. (*n* = 3). Data were obtained from three independent experiments. **p < 0.01.

To elucidate the role of SIRT1 in B[a]P-induced sustained lung inflammation and tumorigenesis, we determined the SIRT1 mRNA and protein levels in BEAS-2B cells after the B[a]P treatment (8 μM). It was shown by RT-PCR and Real-time PCR that the mRNA level of SIRT1 increased gradually upon B[a]P exposure in time-dependent manner (Figure 2A and 2B). The SIRT1 protein level was also induced by B[a]P in a time-dependent manner and reached its peak at 48 h as shown by immunoblotting (Figure 2C). In addition, C57BL/6 mice were exposed to B[a]P and sacrificed after a different period of times (30d, 60d, 90d, 120d, 150d and 180d). The SIRT1 expression in lung biospies was much higher than the controls as shown by immunostain (Figure 2D). Our previous research had demonstrated that B[a]P exposure significantly induced TNF-α expression in mice lung tissues in time-dependent manner [39]. We further detected COX-2 and NF-кB, which played an important role in the inflammatory reaction, on the mice lung biospies. As shown in Figure 2E–2F, the expression of NF-кB increased gradually, while COX-2 increased slightly. Moreover, we detected human lung pathological samples and found that TNF-α, NF-кB and COX-2 were up-regulated in adenocarcinoma and squamous cell carcinoma than normal ones (Figure 2G). We also extended the observation to human lung cancer A549 cells (Figure 2H). SIRT1-luciferase report plasmid was established and evaluated the mechanism of B[a]P induction. It showed that B[a]P induced SIRT1 expression at transcriptional level (Figure 2I).

Taken together, our data strongly demonstrated that B[a]P could up-regulate SIRT1 expression *in vitro* and *in vivo*. We postulated that the up-regulation of SIRT1 might mediate B[a]P-induced chronic inflammation to lung tumorigenesis.

### SIRT1 promoted migration and invasion upon B[a]P exposure

**Figure 3:**
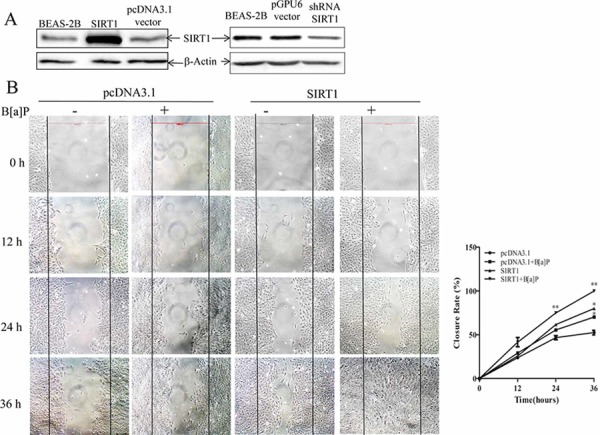

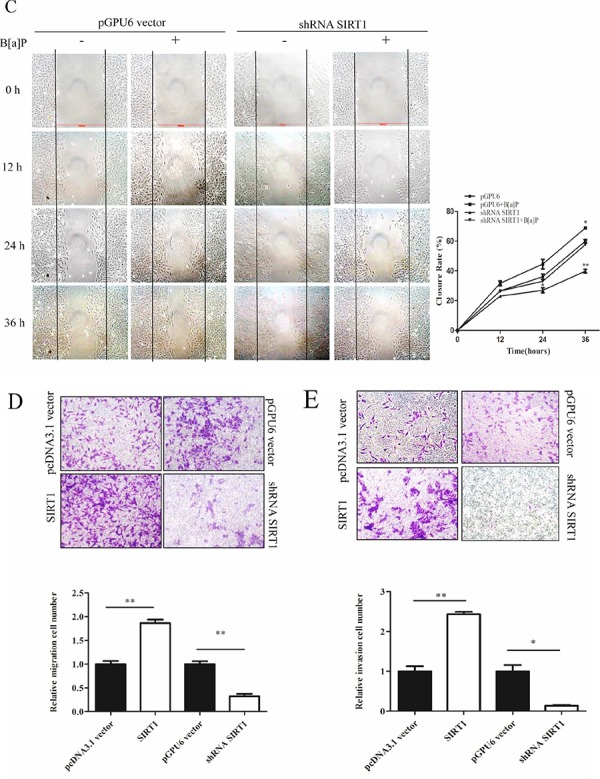
SIRT1 promoted BEAS-2B cells migration and invasion upon B[a]P exposure **A.** BEAS-2B cells were stably transfected with pcDNA3.1 vector/pcDNA3.1-SIRT1 or pGPU6 vector/shRNA-SIRT1. The success of transfection was confirmed by Western blotting. The whole-cell extracts were probed with anti-SIRT1 antibody. **B.** Cell migration behaviors in the stable pcDNA3.1 vector/pcDNA3.1-SIRT1 transfected BEAS-2B cells were evaluated by wound-healing assay. The pictures were taken at different time. **C.** Wound healing assay of pGPU6 vector/shRNA-SIRT1 transfected cells. Quantification(closure rate%) were obtained from three independent experiments and expressed as mean ± S.D. (*n* = 3). *p < 0.05 and **p < 0.01. D–E. Cell migration and invasion ability were analyzed by transwell assay. Results were expressed as mean ± S.D. (n = 3). The numbers of cells were obtained from three independent experiments. **p < 0.01.

Some studies found that SIRT1 might play a role in tumor cell metastasis, such as brain and gastric tumors [4, 40]. Therefore, we performed 3 independant wound healing assays with the BEAS-2B stable transfection cell lines of the empty pcDNA3.1 vector and pcDNA3.1/SIRT1 plasmid (Figure 3A, left). SIRT1 overexpression resulted in an obvious increase in spontaneous wound healing rate as compared to that with the empty vector especially under B[a]P exposure (Figure 3B). Knock-down of SIRT1 by its specific shRNA (compared to pGPU6 vector, Figure 3A, right) attenuated wound healing rate in BEAS-2B cells (Figure 3C). It showed that B[a]P promoted wound healing rate and SIRT1made more obvious effect.

These results indicated that SIRT1 might be involved in cell migration. We also performed the transwell migration assay (filters without Matrigel) and obtained the same results (Figure 3D).

Furthermore, we investigated the effect of SIRT1 on cell invasion using the transwell invasion assay (filters with Matrigel). The invasion of cells through a Matrigel^®^ layer to the underside of a transwell membrane insert was observed (Figure 3E). Our results showed that the cell invasion percentage was enhanced in the SIRT1-overexpressed cell line and reduced in the SIRT1-shRNA cell line in comparison to their corresponding controls. However, without B[a]P treatment, invasion cells were almost zero except in SIRT1 overexperssion cell line (Supplementary Figure S1).

We also detected the cell proliferation and cell death in control BEAS-2B (empty vector), SIRT1-overexpressed cells and SIRT1-shRNA cells. But there were no obvious differences (data not shown). We, therefore, concluded that SIRT1 could be a key mediator of migration and invasion upon B[a]P exposure.

### SIRT1 promoted the expression of TNF-α at transcription level upon B[a]P exposure

TNF-α is the major cytokine involved in inflammatory response, while NF-κB is one of its major regulators [41–43]. Some research reported that SIRT1 was a potent inhibitor of NF-κB signaling and thus suppressed inflammation [25, 44, 45]. It is, therefore, interesting to determine the effect of SIRT1 on the cytokine expression in human bronchial epithelial cells upon B[a]P exposure.

**Figure 4:**
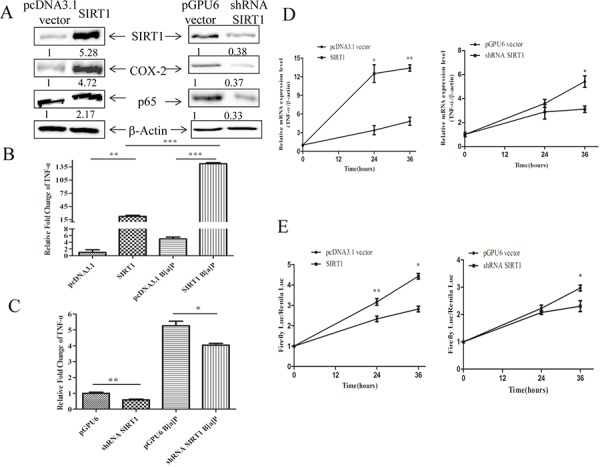
SIRT1 promoted the expression of TNF-α at transcription level **A.** The expression levels of NF-κB and COX-2 were determined in the stable transfection cells of pcDNA3.1-SIRT1 and pGPU6-shRNA by Western blotting. **B.** and **C.** The protein levels of TNF-α were determined in the stable transfection cells of pcDNA3.1-SIRT1 and shRNA-SIRT1 by FACS. The asterisk (**) indicated a significant increase from the medium control cells (*p* < 0.01). **D.** The mRNA levels of TNF-α in the stable transfection cells of pcDNA3.1-SIRT1 or shRNA-SIRT1 determined by RT-PCR. **E.** The transcription activities of TNF-α determined by dual luciferase report system. The asterisk (*) indicated a significant induction from B[a]P-treatment (*p* < 0.05).

Our results showed that SIRT1 overexpression enhanced the expression of NF-κB and COX-2, whereas silencing SIRT1 reduced the levels of NF-κB and COX-2 in BEAS-2B cells by Western blotting (Figure 4A). As shown in Figure 4B and 4C, the overexpression of SIRT1 could increase the level of TNF-α, while silencing SIRT1 reduced its expression as determined by FACS. To further test our hypothesis, the stable transfection cells of BEAS-2B cells and their control cells were treated with 8 μM B[a]P for different times (0,12,24,36 h). SIRT1 overexpression facilitated the induction of TNF-α mRNA, while knock-down of SIRT1 reduced it (Figure 4D).

To determine whether SIRT1 enhanced the TNF-α expression at the transcriptional level, we measured the TNF-α promoter activity in the SIRT1 overexpressing or SIRT1 silencing cells upon the B[a]P exposure. TNF-α transcriptional activity were elevated in the SIRT1 overexpressing cells upon B[a]P treatment, but reduced in the SIRT1 silencing cells (Figure 4E). Thus these results showed that SIRT1 mediated B[a]P-induced TNF-α expression at the transcription level.

### TNF-α acted downstream of SIRT1 to promote cell migration and invasion

**Figure 5:**
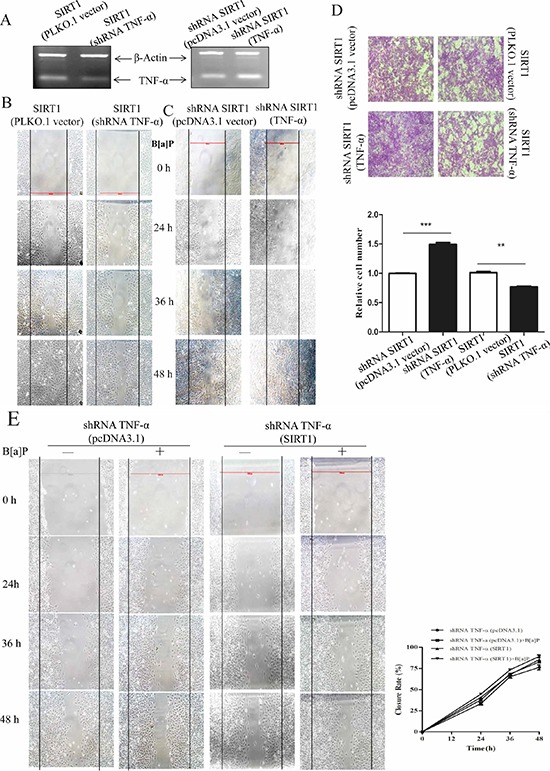
TNF-α played a role in SIRT1 promoting BEAS-2B cell migration and invasion upon B[a]P exposure **A.** The levels of TNF-α mRNA were detected by RT-PCR in the SIRT1 (PLKO.1 vector)/SIRT1 (TNF-α silencing) or SIRT1 silencing (pcDNA3.1 vector)/SIRT1 silencing (TNF-α) transfected cells. **B.** and **C.** Cell migration behaviors of the above cells were tested by wound-healing assay. **D.** Cell invasion ability of the above cells were tested by transwell assay. **E.** Comparing the cell migration behaviors of TNF-α silencing (pCDNA3.1/pcDNA3.1-SIRT1 and pGPU6/shRNA-SIRT1) cells with or without B[a]P treatment by wound-healing assay. Results were expressed as mean ± S.D. (*n* = 3). The numbers of cells were obtained from three independent experiments.

TNF-α is an important inflammatory factor and has been implicated in the migration and invasion [27, 46–49]. In order to assess the functional contributions of TNF-α in cell migration and invasion, we transfected TNF-α shRNA into the SIRT1 overexpression cells (Figure 5A) that were subject to wound healing assay and transwell assay. We also overexpressed TNF-α in SIRT1 silencing cells, and found TNF-α could accelerate wound healing compared to the controls (pcDNA3.1 vector only, Figure 5B and 5C). TNF-α silencing could eliminate the effects of SIRT1 on the invasion ability of BEAS-2B cells (Figure 5D). Furthermore, we compared the difference among control cells, SIRT1 overexpressed cells with or without B[a]P treatment by wound healing assay under the conditions of silencing TNF-α. It showed no significant difference (Figure 5E).

Based on the above results, we proposed that TNF-α acted downstream of SIRT1 to promote BEAS-2B cell migration and invasion upon B[a]P exposure.

### The crosstalk existed between TNF-α and Wnt/β-catenin signaling

**Figure 6:**
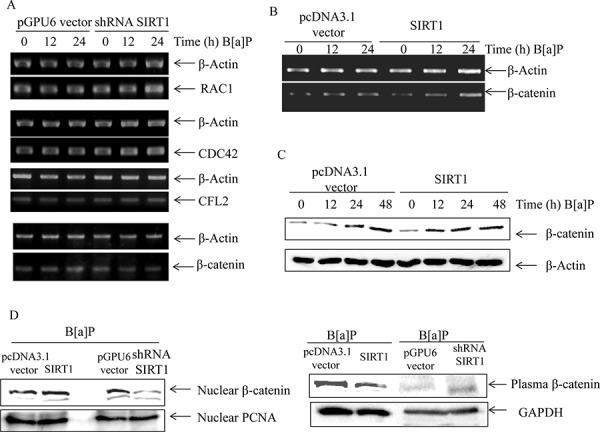
SIRT1 promoted β-catenin accumulation upon B[a]P exposure **A.** The expression of the invasion-associated genes (Ras-related C3 botulinum toxin substrate 1, RAC1/Cell division cycle 42, CDC42/Cofilin2, CFL2/β-catenin in the pGPU6 vector/pGPU6-SIRT1 transfected cells under B[a]P exposure were examined by RT-PCR. **B.** The expression levels of β-catenin mRNA in the pcDNA3.1 vector/pcDNA3.1-SIRT1 transfected cells were detected by RT-PCR. **C.** The stable transfection cells of pcDNA3.1-SIRT1 and its vector were exposed to B[a]P as indicated. The protein levels of β-catenin were determined by Western blotting. **D.** The nuclear and plasma levels of β-catenin in SIRT1 overexpression, SIRT1-shRNA and their comparing vectors were determined by Western blotting.

In order to indentify the key mediators in cell migration and invasion, we first screened some important factors of cytoskeletal reorganization and motility in SIRT1 silencing cells by RT-PCR. As shown in Figure 6A, we found that SIRT1 silencing did not reduce the mRNA levels of RAC1/CDC42/CFL2/XIAP, except β-catenin, upon B[a]P exposure. To confirm the results, we used the SIRT1 overexpression cells and its controls and obtained the similar results (Figure 6B). In consistent with β-catenin mRNA, β-catenin protein level was also up-regulated in SIRT1 overexpression cells (Figure 6C). Furthermore, we extracted nuclear and plasma protein to evaluate the nuclear translocation of β-catenin. As shown in Figure 6D, nuclear β-catenin variation was opposite to plasma β-catenin. It suggested that SIRT1 could promote the nuclear translocation of β-catenin.

In short, the results showed that SIRT1 induced β-catenin expression in both protein and mRNA levels in time-dependent manner. SIRT1 could stimulate its nuclear accumulation.

**Figure 7:**
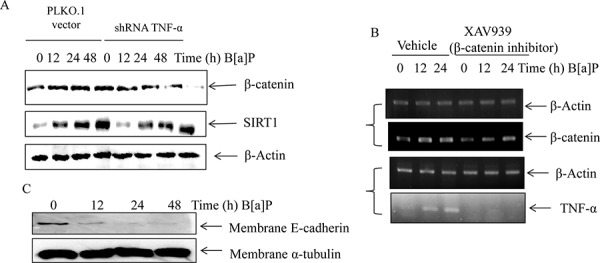
The crosstalk existed between TNF-α and Wnt/β-catenin signaling in B[a]P-treated BEAS-2B cells **A.** The protein levels of β-catenin in the PLKO.1 vector/PLKO.1-TNF-α silencing transfected cells under B[a]P exposure were examined by Western blotting. **B.** The mRNA levels of TNF-α in BEAS-2B cells after XAV939 (1 μM) treatment for 24 h by RT-PCR assay. C. BEAS-2B cells were treated with 8 μM B[a]P for 48 h. The cellular lysates were fractionated and the protein expression levels of E-Cadherin were examined by Western blotting.

Moreover, we studied interaction between TNF-α and β-catenin by TNF-α silencing and Wnt/β-catenin specific inhibitor XAV939. TNF-α silencing could reverse the effects of SIRT1 on the β-catenin protein expression in BEAS-2B cells upon B[a]P exposure (Figure 7A). On the other hand, the mRNA level of TNF-α decreased under XAV939 treatment (Figure 7B). These results indicated that TNF-α and Wnt/β-catenin signaling could have crosstalk in B[a]P-treated BEAS-2B cells.

Most studies found that E-cadherin was an important adhesion membrane protein to regulate cell migration and invasion. The E-cadherin expression was regulated by Wnt/β-catenin signaling pathway and significantly reduced in the majority of metastatic carcinomas. Thus we determined its expression level by cell fractionation and western blot in BEAS-2B cells after B[a]P exposure and found that it was markedly reduced (Figure 7C). These results suggested the B[a]P exposure increased the migration and invasion of BEAS-2B cells through decreasing the adhesion molecule expression level.

### SIRT1 induced tumorigenesis of BEAS-2B cells after chronic B[a]P treatment

**Figure 8:**
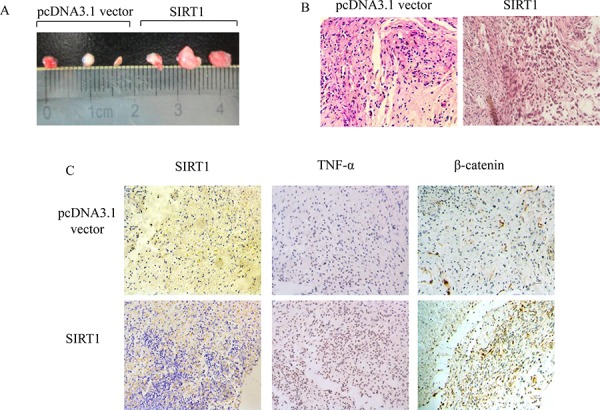
SIRT1 induced tumorigenesis of BEAS-2B cells after chronic B[a]P treatment The pcDNA3.1 vector and pcDNA3.1-SIRT1 transfected BEAS-2B cells were repeatedly treated with 8 μM B[a]P for 8 weeks. Cells were injected s.c. into nude mice at 2 × 10^6^ each spot. **A.** The pictures of the tumors from the SIRT1 overexpression and control BEAS-2B cells in nude mice. **B.** The pathological pictures of the SIRT1 overexpressing tumors were observed and photographed under microscope (×400). **C.** Immunohistochemical staining of SIRT1/TNFα/β-catenin was performed on (×400).

In order to further clarify the role of SIRT1 in tumorigenesis, we carried out nude mice tumorigenicity assay. The SIRT1-overexpressing cells and its control cells were treated with 8 μM B[a]P for 8 weeks. The cells were harvested and injected subcutaneously (s.c.) into nude mice at three spots with 2 × 10^6^ cells each. Eight weeks later, the tumors were counted and measured. The results showed that, compared to the control cells, the tumor volume of the SIRT1-overexpressing cells with long term repeated exposure to B[a]P was significantly larger (Figure 8A and 8B). Meanwhile, the expression levels of TNF-α and β-catenin in the SIRT1 overexpression group were significantly higher than the control group (Figure 8C). Therefore, SIRT1 could stimulate the tumorigenicity of BEAS-2B cells upon B[a]P exposure in nude mice through TNF-α/β-catenin pathways.

## DISCUSSION

Cigarette smoke and vehicle exhaust contain a high concentration of B[*a*]P that has been proved to be associated with lung cancer [50, 51]. Although there are many cytokines contributing to lung cancer development and progression, the underlying molecular mechanisms is not well understood.

SIRT1, the founding member of the class III HDACs, uses NAD^+^ to mediate the deacetylation of histone and non-histone proteins [52]. SIRT1 plays a dual role in cancer development and progression. The SIRT1 expression is significantly downregulated in human head and neck squamous cell carcinoma (HNSCC). And the high SIRT1 expression is associated with a good prognosis [53]. SIRT1 transgenic mice exhibit reduced susceptibility to carcinogen-induced liver cancer [54]. SIRT1 overexpression in ApcMin/+ mice induces beta-catenin deacetylation, reducing colon tumor formation [55]. On the other hand, it recently has been implicated in the initiation and progression of various malignancies [56]. SIRT1 can promote cell migration and metastasis by directly interacting and deacetylating cortactin in breast cancer [57]. SIRT1 could stimulate tumor growth by increasing vessel density and downregulating DLL4/Notch signaling in lung cancer [58].

Our study showed that the B[a]P treatment could induce SIRT1 expression in BEAS-2B cells and animal lung samples. And the SIRT1 overexpression was also detected in human lung cancer biopsies, both in adenocarcinoma and squamous cell carcinoma. The cell migration and invasion is one of the most important hallmarks for cancer cells [59]. We also illustrated that SIRT1 could stimulate the migration and invasion in B[a]P-exposed BEAS-2B cells. More important, the SIRT1 overexpression could obviously promote tumorigenesis in nude mice while SIRT1 silencing could inhibit the process. It strongly suggested that SIRT1 might play an important role in lung tumorigenesis.

To elucidate the possible mechanisms of SIRT1 elevation upon B[a]P exposure, We screened some potential factors, such as AP1, NF-кB and Sp1 *et al*., and found that AP1 might be responsible for SIRT1 elevation. However, it need more evidence.

The chronic inflammation promotes lung carcinogenesis. Several transcription factors, enzymes and cytokines were involved in the process [60–62]. Tumor necrosis factor (TNF-α) is a major cytokine involved in inflammation, immunity, cell migration/invasion and tumorigenesis. It is one of the important regulatory factors to stimulate cancer-related inflammation [63–66]. Our data showed that TNF-α was induced in B[a]P treated mice and was associated with lung tumorigenesis. SIRT1 overexpression increased TNF-α promoter activity induced by B[a]P in BEAS-2B cells, while SIRT1 silencing reduced it. We also found that knock-down of TNF-α could eliminate the effect of SIRT1 on invasive and migratory ability of BEAS-2B cells. It suggested that TNF-α was the down-stream effector in B[a]P-induced cell transformation.

**Figure 9:**
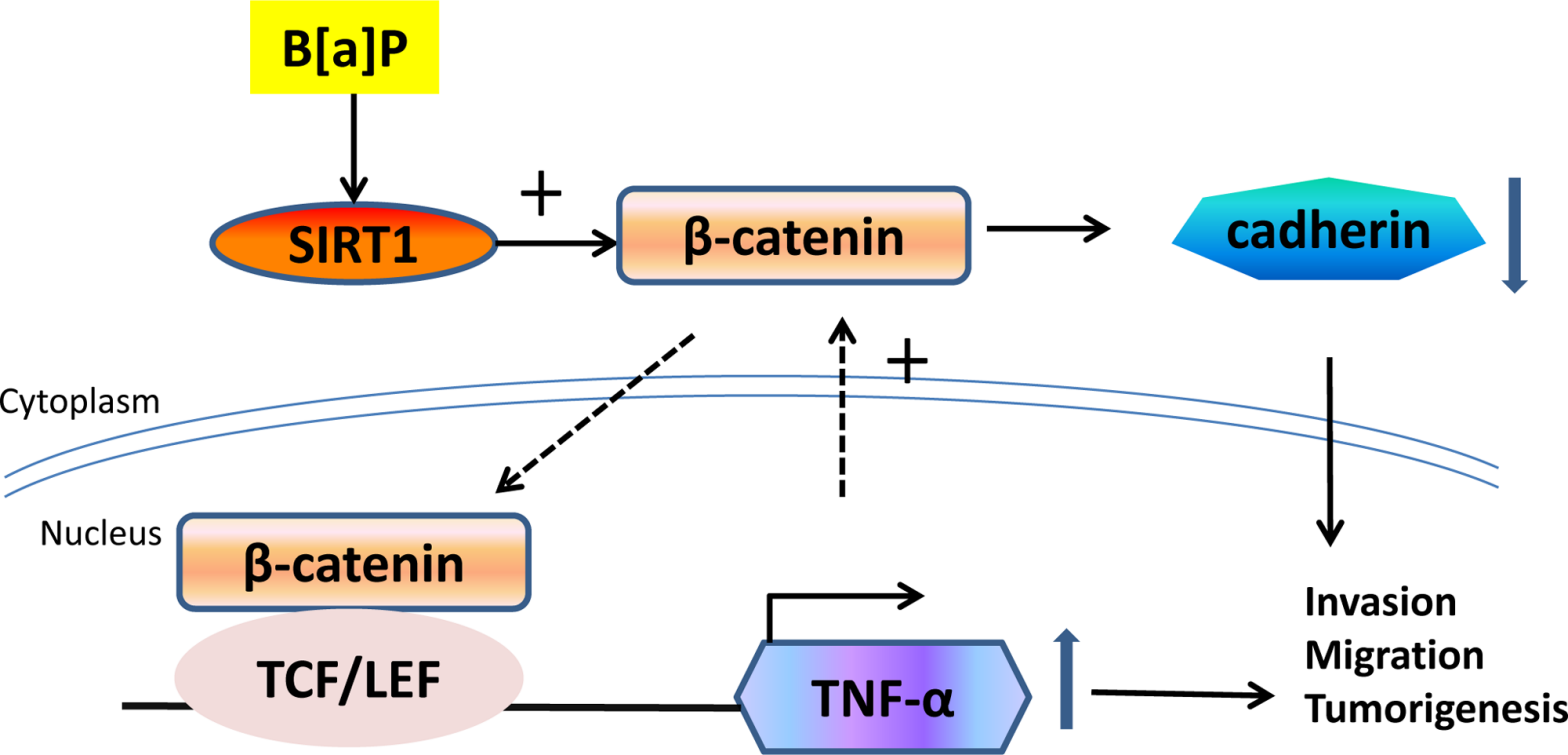
The proposed scheme depicts the roles of SIRT1 in B[a]P-induced inflammation and tumorigenesis Shown was the schematic representation of the underlying molecular mechanisms of B[a]P induced inflammation and tumorigenesis. SIRT1 was induced by B[a]P exposure and enhanced β-catenin accumulation. β-catenin translocated into nucleus and activated TNF-α transcription. TNF-α increase somehow enhanced β-catenin expression that in turn amplified the effect of SIRT1 and led to sustained inflammation and lung cancer development.

It has been reported that many transcription factors could bind to the TNF-α promoter, such as NF-кB and AP1 [67]. In this work, we found that β-catenin was up-regulated at the transcription level in a SIRT1-dependent way. XAV939, a Wnt/beta-catenin specifice inhibitor, reduced the mRNA levels of TNF-α, while β-catenin stimulated the promotor activity of TNF-α. On the other hand, silencing TNF-α by shRNA inhibited the expression of β-catenin. It indicated that there was a positive feedback between the TNF-α and Wnt/β-catenin signaling pathways and amplified the effect of B[a]P (Figure 9). Epithelial-mesenchymal transition (EMT) is a key step toward cancer progression, invasion and metastasis [68]. A common hallmark of EMT is the breakdown of E-cadherin expression or function [69]. Therefore, we surmised that SIRT1 could drive EMT to tumorgenesis.

In conclusion, SIRT1 could contribute to the development of lung cancer through TNF-α/β-catenin axis. Our study demonstrated that SIRT1 was one of the key regulators in B[a]P-induced lung tumorigenesis and would be a potential therapeutic target for lung cancer intervention.

## MATERIALS AND METHODS

### Cell cultures

BEAS-2B cells and its stable transfection cell lines were cultured in Dulbecco's modified essential medium (DMEM) (GIBCO, USA) supplemented with 10% fetal bovine serum (FBS) (GIBCO, USA), 100 IU/ml streptomycin, 100 IU/ml penicillin and 2 mM L-glutamine (GIBCO, USA) at 37°C in a humidified atmosphere with 5% CO_2_. B[a]P exposure: BEAS-2B cells were treated with 8 μM B[a]P (Sigma-Aldrich, USA). Wnt/β-catenin specific inhibitor: BEAS-2B cells were treated with 1 μM XAV939 (Sigma-Aldrich, USA) for 24 h.

### Transfection

To overexpress and knock down SIRT1 in BEAS-2B cells, the cells were transfected with pcDNA3.1/SIRT1 plasmid or pcDNA3.1 vector as a control, and pGPU6/shSIRT1 plasmid or pGPU6 vector as a control (GenePharma, China), respectively, using Lipofectamine 2000 as manufacturer instructed (Invitrogen, USA). For stable transfection, cultures were subjected to G418 or puromycin drug selection, and survived cells from the antibiotic selection were pooled as stable mass transfectants. These stable transfectants were then cultured in the selected antibiotic-free medium for at least two passages before being used for experiments. The stable transfections obtained from the transfection mentioned above are named as, respectively. Two days after transfection, the cells were cultured with G418 (GIBCO, USA) or puromycin (Invitrogen, USA) to establish stable transfection. The established stable transfection cells were cultured in G418-free or puromycin-free 10% FBS DMEM for at least two passages before each experiment.

### Western blotting

When BEAS-2B cells and its stable transfection cells were cultured in 6-well plates to 50∼60% confluence, the culture medium was replaced with 1% FBS DMEM. After 12 h, the cells were exposed to 8 μM B[a]P for indicated time. Cells were then lysed in 1 × cell lysis buffer plus 1 × phenylmethanesulfonyl fluoride (PMSF). Cellular lysates were centrifuged at 12,000 rpm for 15 min at 4°C, and then separated by sodium dodecylsulfate polyacrylamide gel electrophoresis (SDS-PAGE). Proteins were blotted onto polyvinylidene difluoride (PVDF) membrane (Bio-Rad, USA) that was probed with each of the following antibodies against β-Actin (Beyotime Institute of Biotechnology, China), SIRT1, β-catenin, COX-2 (Santa Cruz, USA). The protein bands specifically bound by the primary antibodies were detected using horse reddish peroxidase-linked secondary antibody and ECF (Beyotime Institute of Biotechnology, China).

### RT-PCR and real-time PCR

RNA was reverse transcripted to cDNA with PrimeScript™ RT reagent Kit (Perfect Real Time) (Takara, Japan), then the cDNA was used as the template for RT-PCR to examine SIRT1 mRNA. The amplification cycles were 28. The human β-Actin gene was used as internal control. The primers used in RT-PCR were listed as below:

5′-TTCAGGTCAAGGGATGGTATTT-3′ (forward) and 5′-TGTTCCAGCGTGTCTAAGTTCT-3′ (reverse) for SIRT1

5′-CGGTACAACGAGCTGTTTCTAC-3′ (forward) and 5′-AGCTTCCAGACACGCTATCAT-3′ (reverse) for β-catenin

Real-time PCR with SYBR Green PCR Master Mix (Applied Biosystems, Foster City, CA) was performed using ABI Prism 7700 Sequence Detector (Applied Biosystems). First strand cDNA and real-time PCR were performed as previously described. The primers for TNF-α used in Real-time PCR were:

5′-TCTTCTCATTCCTGCTTGTGG-3′ (forward) and 5′-CACTTGGTGGTTTGCTACGAC-3′ (reverse).

### Cell migration and invasion assay

According to manufacturer's instruction, the cell invasion and migration assays were performed using a transwell membrane (Corning, USA). In the invasion assay, Matrigel (BD Biosciences, USA) was applied to the upper chamber. BEAS-2B stable transfection cell lines, pretreated with B[a]P (8 μM) for 48 h, were trypsinized and seeded at 5 × 10^4^ cells per insert (in triplicates) in 100 μL serum-free DMEM medium. Inserts were placed in 600 μL DMEM medium with 10% FBS. The cells were incubated for certain time (migration assay for 24 h/invasion assay for 48 h) in an incubator with 5% CO2 humidified atmosphere. Then the cells on the upper surface of the filters were completely removed by wiping with a cotton swab. The inserts were fixed in 4% polyfluoroalkoxy (PFA) for 30 min and stained with crystal violet staining solution (Beyotime Institute of Biotechnology, China). The picture acquisition was done under an Olympus microscope (Olympus Corporation, Japan) and images were taken at 200× magnification with the same light intensity and exposure time. For invasion assay, the numbers of cells attached to the other side of the insert were counted under a light microscope in 8 random fields at a magnification of ×200. For migration assay, after photographed, the cells migrated to the underside were eluted by 33% acetic acid, and the cell numbers were measured under an Olympus microscope or by VarioskanFlash (Thermo, USA) at 570 nm. The data shown were representatives of three independent experiments.

### Cell wound healing assay

Cells were seeded into 6-well tissue culture plates and grown to 90% confluence as a monolayer. The monolayer was scratched vertically with a new pipette tip across the center of the wells. After washing with PBS three times, the cells were continually cultured with 3% FBS medium plus 8 μM B[a]P for indicated times. Photos were taken every 12 h until the wound healed in the parental cells.

### Flowcytometric analysis (FACS)

Cells were stimulated with 8 μM B[a]P for 48 h and used protein transport inhibitor, containing Brefeldin A (BD, USA), six hours ahead of the end of reaction. Then cells were harvested, and incubated with 1× Fixation/Permeabilizaton Diluent (eBioscience, USA) overninght at 4°C. Cells were washed and stained with PE-conjugated anti-TNF-α (Biolegend, USA) for 3 h at 4°C. FACS assays were performed and analyzed using flow cytometer (Beckman Coulter, USA).

### Luciferase reporter assay

Cells were transfected with the indicated luciferase reporter. Relative Firefly luciferase activity to TK Renilla (internal control) was measured with dual-luciferase reporter assay system (Promega, USA) in an automatic microplate reader (Thermo, USA).

### Collection of lung tissue clinical samples

Lung tissue samples were acquired from the First Affiliated Hospital of Wenzhou Medical University, Wenzhou. The collection was approved by the University Ethic Committee, and written informed consent was obtained from each patient. The patients were recruited based on pathological diagnoses of lung cancer. There was no evidence of any other malignancies and no history of preoperative anticancer treatment. After each surgical removal, the lung tissue samples were immediately snap-frozen in liquid nitrogen and stored at −80°C until subsequent analysis.

### Animal experiments

Wild-type C57BL/6 female mice were kept in SPF animal facility at Wenzhou Medical University and were randomly divided into the treatment and control groups. Each animal was exposed to B[a]P (dissolved in 50 μl tricaprylin solvent) by intratracheal instillation at 1 mg/mouse, while the control animal received the same amount of tricaprylin solvent only. The treatment was repeated once a week for 4 weeks. The concentrations and timings of the intratracheal instillation of B[a]P were chosen based upon lung tumor induction by B[a]P in previous studies, and were adjusted, if necessary, to cause maximal induction of SIRT1 expression with minimal observed toxicity to mice. Five mice each from the B[a]P treatment and tricaprylin control groups were sacrificed on days 1, 30, 60, 90, 120, 150 and 180 after last exposure, and 30 mice from each group were sacrificed on day 180 after exposure to evaluate the effect of B[a]P on SIRT1 expression.

### SIRT1 immunohistochemistry

The clinical and animal lung samples were collected as described above. Immunohistochemistry was conducted as described [70, 71]. Briefly the lung tissue paraffin sections were heat treated at 65°C, deparaffinized with xylene and rehydrated through serial concentrations of ethanol. After antigen retrieval, the slides were quenched by hydrogen peroxide in PBS and blocked with 20% sheep albumin. Then they were incubated with a rabbit anti-SIRT1 antibody (Abcam, USA) (1:200 dilution in PBS) overnight at 4°C, followed by rinsing three times with PBS, and incubated for 1 hour at room temperature with HRP-conjugated goat anti-rabbit antibody (Beyotime Institute of Biotechnology, China) at 1:200 dilution in PBS. After being washed three times in PBS and incubated with 3′3-diaminobenzidine tetra-hydrochloride (DAB), the slides were counterstained with haematoxylin and finally mounted with neutral balata. Negative controls without the primary antibody incubation were also performed in the same way as described above.

The picture acquisition was done under an Olympus microscope (Olympus Corporation, Japan) and images were taken at 200× magnification with the same light intensity and exposure time. The SIRT1 staining intensity of lung tissues was semi-quantitatively measured by the Image-Pro Plus image analysis software (Media Cybernetics, USA) as the integrated optical density (IOD) value of each image. IOD was obtained from total lung (mixing bronchi and parenchyma).

### BEAS-2B tumorigenic abilities in nude mice

BEAS-2B pcDNA3.1 vector and SIRT1 overexpression cells were treated with 8 μM B[a]P for 3 days. The cultures were split 1:10 and then treated for a second time with B[a]P. This treatment was repeated for 7 weeks. The cells were harvested and the nude mice were injected subcutaneously (s.c.) in three spots with 2 × 10^6^ of B[a]P-treated BEAS-2B pcDNA3.1 vector or SIRT1 overexpression cells in PBS. Eight weeks later, the tumors were dissected and measured.

### Statistical methods

Student's *t*-test was used to statistically assay the significance of differences of mRNA levels, relative fluorescence intensities, and immunohistochemical signals, etc. The difference was considered significant at *p* < 0.05.

## SUPPLEMENTARY FIGURE


