# Supplementary material for: SIRT1 in B[a]P-induced lung tumorigenesis
Source: Oncotarget. 2015 Aug 4;6(29):27113–29. doi: 10.18632/oncotarget.4729 (PMC4694977; doi:10.18632/oncotarget.4729)
Supplement: Supplementary file 1 [file oncotarget-06-27113-s001.pdf]

SUPPLEMENTARY FIGURE

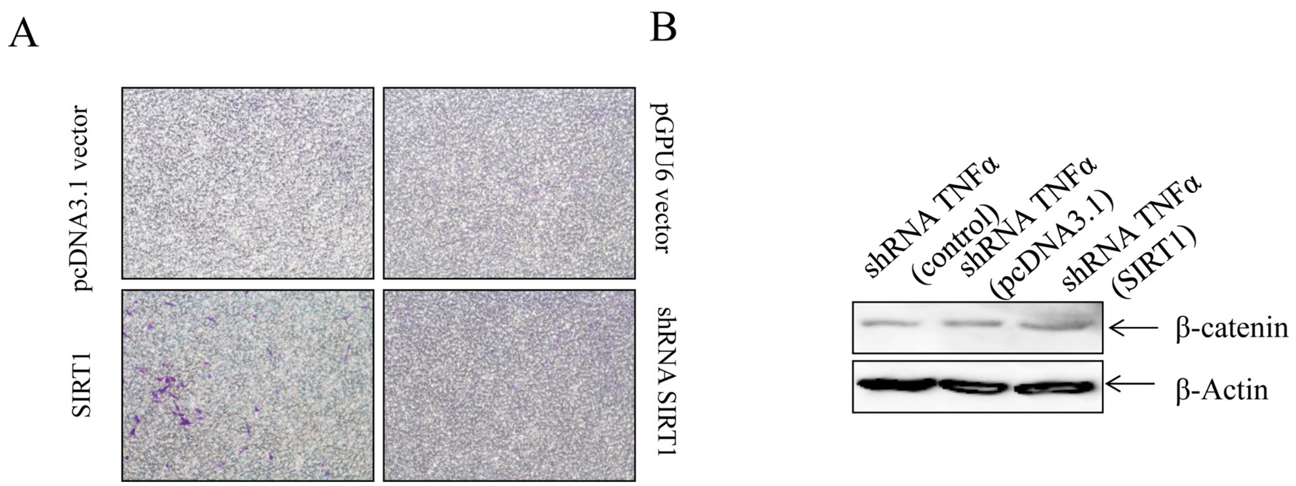

Supplementary Figure S1: A. Invasion ability of the BEAS-2B cells stably transfected by pcDNA3.1 vector/pcDNA3.1-SIRT1 or pGPU6 vector/shRNA-SIRT1 was analyzed by transwell assay without B[a]P treatment. **B.** The expression levels of β-catenin in TNFα-silencing (control/pCDNA3.1/pcDNA3.1-SIRT1) cells were examined by Western blotting.
